# Conference Report: WORKSHOP ON CHARA CTERIZING DIAMOND FILMS III Gaithersburg, MD February 24–25, 1994

**DOI:** 10.6028/jres.099.026

**Published:** 1994

**Authors:** Albert Feldman, Sandor Holly, Claude A. Klein, Grant Lu

**Affiliations:** National Institute of Standards and Technology, Gaithersburg, MD 20899-0001; Rockwell International, 6633 Canoga Ave., Canoga Park, CA 91304; C.A.K. Analytics, 9 Churchill Lane, Lexington, MA 02173; Norton Diamond Film, Goddard Rd., Northboro. MA 01532

## 1. Introduction and Conclusions

The third in a series of workshops was held at NIST on February 23–24, 1994, to discuss, in depth, specific topics deemed important to the characterization of diamond films made by chemical vapor deposition (CVD diamond) and to address the need for standards in diamond technology. The topics chosen for this workshop were based on feedback from the attenders of the previous workshop. The audience targeted for this workshop consisted of producers and potential users of CVD diamond technology. University scientists and scientists from government laboratories were invited as experts in properties measurements. There were 55 attenders at the workshop.

We focussed on three technical topics for discussion: characterizing brazing and polishing, standardization of thermal conductivity measurements, and characterizing stress strain, and fracture.

The principal conclusions of the workshop include:

- The development and availability of reliable post growth processes, such as polishing and brazing, will contribute in a substantial way to rcalizing new applications of CVD diamond. It is important to characterize and evaluate the results of these processes in a uniformly accepted way.
- The first results of the interlaboratory round-robin comparison of thermal conductivity and thermal diffusivity measurements were presented. They showed considerable laboratory to laboratory variations, although much of this variability may be due to the manner in which the different measurement methods employed are affected by specimen inhomogeneity and specimen anisotropy. Additional laboratories will be making measurements that will add to the present measurement data base.
- CVD diamond exhibits lower resistance to fracture than other forms of diamond. This is impeding the use of CVD diamond in applications where the material is subjected to high stresses, such as those induced by thermal shock. The strength deficiency is attributed to surface flaws and high internal stresses that originate during growth. The relationship between growth conditions and internal state of stress has not yet been resolved.

## 2. Characterizing Brazing and Polishing

There were six presentations in this session. The first three presentations centered on techniques for polishing and surface figuring CVD diamond. The last three talks discussed issues and techniques for bonding diamond to other materials.

The development and availability of reliable post-growth processes, such as polishing and brazing, will contribute in a substantial way to realizing new applications of CVD diamond. Being able to characterize and evaluate the results of these processes in a uniformly accepted way is very important. The quality of a brazed joint between a CVD diamond component and a ceramic or metal support structure may be defined in a number of different ways, depending on the specific application. In one application the most important feature of a joint might be its heat transfer characteristics at cryogenic temperatures or across a broad temperature range; in another application its most important characteristic may be its mechanical strength, which might be determined by the level of success in compensating for the thermal expansion mismatch between the substrate and the diamond. Optical applications will be driven by other requirements, such as how a specific polishing process controls the surface micro-roughness or how closely an accurate surface figure can be attained.

The first presentation was by R, Miller of Raytheon who gave an extensive overview of diamond polishing technology. In his talk, he grouped the currently pursued polishing methods into contact type and noncontact type processes. Contact methods may be further divided into nonreactive and reactive methods. The non-reactive polishing methods discussed were the conventional and the abrasive jet techniques. Among the reactive polishing techniques, the hot metal polishing approach was emphasized. Polishing diamond against iron, manganese, lanthanum, and cerium metals has had variable success.

The iron plate method is usually a dynamic method in which the diamond is rubbed against the iron at an elevated temperature. It results in a smooth finish (20 nm peak-to-valley) when the plate temperature is low (≈750 °C) and the polishing is done in a hydrogen environment; the material removal rate is relatively low (≈0.5 μm/h). The polishing process and the material removal rate may be speeded up (≈7 μm/h) by increasing the plate temperature to ≈950 °C. However, this results in a rough finish. An appropriate sequence of polishing temperatures appears to result in an acceptably smooth surface in a reasonable length of time. However some intergranular cracks may form during the processing which may or may not be acceptable, depending on the application. A method for characterizing the cracks in the polished CVD diamond surface must be used in order to evaluate the seriousness of such cracks.

Molten lanthanum or cerium metals, used in a static procedure, minimize the intergranular cracking in CVD diamond during polishing. In this procedure, the diamond is placed in contact with the metal, but no relative motion takes place, that is, no rubbing occurs. This process results in higher material removal rates than the previous method, but at the cost of increased surface roughness.

Other reactive contact polishing methods mentioned were float polishing employed at the Naval Air Warfare Center and amorphous silicon oxide techniques used by Edge Technologies, Inc.

Noncontact polishing methods fell into two groups, those that use laser beam etching and those that use ion beam etching. Polishing with a YAG laser beam at either 1.06 μm or at 0.53 μm and with an excimer laser beam was discussed. Two different ion beam techniques have been used to polish of CVD diamond. In the first technique, diamond is removed by sputter-etching with an argon or oxygen ion beam. A second novel technique has been developed by the Spire Corporation. It uses ion implantation to soften a top layer of diamond about 1 μm thick, which is then removed by conventional polishing methods.

The second talk was given by T, S, Sudarshan of Materials Modification Inc. He summarized recent advances in the use of lasers for polishing diamond films. Special emphasis was placed on the difficulty associated with polishing large areas and on the interpretation of the characterization of the polished diamond surfaces. Technical areas in which surface roughness plays an important role, such as tribological applications, were discussed.

The last talk on polishing was presented by S. Holly of Rockwell International, Rocketdyne Division. He highlighted the importance of surface shape control during polishing. Several applications require polished surfaces that are very flat. These include thermal applications, such as large area diamond plate heat-sinks for multichip module technology and optical applications such as infrared windows. Future applications of CVD diamond will require accurate control of non-flat surfaces, such as spherical, aspherical, cylindrical, and toroidal shapes, that might be used in optical domes and lenses.

A polishing procedure that utilizes a combination of several polishing methods was then described. The procedure is based on interferometrically controlled ablation of diamond to produce a flat surface. The material removal proceeds in a manner similar to that used in single-point diamond turning; however, in this process, material removal is controlled in real time by continuous interferometric monitoring of the surface being ablated. The present status and recent results of this polishing procedure were discussed.

The first talk on CVD diamond brazing was presented by T P. Thorpe of the Naval Research Laboratory (NRL). He focused on the importance of reliable brazed joints for producing high quality homoepitaxial diamond. These joints provide a thermal path between the diamond substrate and an underlying heat sink. The joints must survive high temperatures (up tp 1500 °C) for extended periods of time. Because the temperatures are so high, the conventional commercial brazing material (from Drukker International), a gold-tantalum composite which melts at ≈ 1060 °C, could not be used. As a result, several alternative brazes were investigated.

For NRL’s purposes the selection criteria for a braze material were; 1) it should contain a refractory carbide-forming element in order to achieve satisfactory bonding to the diamond substrate. 2) It should form a refractory compound or alloy with the underlying molybdenum heat sink. 3) All products created during braze formation should have a melting point above 1300 °C. Among the brazes tried were several variations on the Drukker braze recipe, including substitution or alloying of gold with different members of the platinum group of metals. Greatest success was achieved with a Pd substitution. This was primarily due to its relatively low melting point of 1552 °C which helped to minimize graphitization of the diamond surface during braze formation. Platinum-gold alloys were also found to be effective but they were inherently more difficult to fabricate due to an unexpectedly complex Au-Pt interaction. Both of the above mentioned brazes were found to withstand growth conditions for several hours at temperatures up to 1400 °C without degradation.

Diamond-metal brazes employing Nichrome were also fabricated. They were able to withstand temperatures up to 1200°C. Details of all of the fabrication techniques were discussed.

The second talk on brazing was given by R. Meilunas of the Grumman Corporate Research Center. The talk emphasized the importance of rigorous joint design procedures. A practical approach to braze design would include: 1) Consider the temperature of operation of the joint; select the melting point of the braze alloy to be 100 °C to 200 °C above the operation temperature. 2) Select the joint geometry. 3) Determine the mechanical properties of each component to be brazed. 4) Determine the thermoplastic/thermoplastic properties of the braze alloy (such as stress/strain vs temperature, strain hardening coefficient, elastic modulus, Poisson’s ratio). 5) Determine the welting/bonding properties of the braze alloy to the components to be brazed, 6) Perform a stress analysis of the joint, 7) Perform experimental validation of the stress analysis. 8) Determine the environmental stability of the joint.

A list of practical braze alloys was presented that covered brazing temperatures between 280 °C and 1300 °C. The properties of several substrate materials, such as W, Be and Cu, were related to the brazing process. The value and importance of a finite element analysis of brazed joints was described. The following statements concluded the presentation: 1) The stability of diamond-metal joints depends critically upon the residual stresses developed during cool-down from the brazing temperature. 2) A finite element stress analysis can provide insights into the residual stress distribution and potential failure modes in the joints. 3) The design of CVD diamond-metal brazed joints requires knowledge of wetting, chemical bonding, and metallurgical interactions during brazing.

The final talk of the session was given by J. Intrater, Oryx Technology Corporation. The talk focused on specimen surface preparation and selection of methods for analyzing the brazed joint. In considering sample surface preparation, special emphasis was placed on the challenges of sectioning and polishing of diamond and the problems that arise from the vast differences in hardness between metals and diamond. Important to characterization of metallization on diamond is the determination of the elemental distribution throughout the interfacial region and an assessment of the carbon phases present (i.e., the amount of *sp*^2^ and *sp*^3^ carbon present). The talk discussed use of a scanning electron microscope (SEM) equipped with an x-ray microprobe and a wavelength dispersive spectrometer and Raman spectroscopy for evaluating the joint.

## 3. New Developments

In order to foster international cooperation in the development of standards for CVD diamond, we invited Yoichiro Sato of the National Institute for Research in Inorganic Materials (also known as NIRIM) to discuss the need for standardized methods of characterization and property measurement in Japan. In the area of CVD diamond, no standards development is occurring in Japan at present. Dr. Sato expressed the view that the large property variations that occur in CVD diamond make standards development difficult at this time. He has agreed to inquire whether other laboratories in Japan might want to participate in the round robin measurements of thermal conductivity.

K. V. Ravi of Lockheed Missiles & Space Company discussed the optical transmission properties of diamond produced by a novel combustion flame method. Some in the audience questioned whether the transmissivity was as good as was claimed, based on certain features seen in the absorption spectrum. This issue was not resolved.

E. Etz of NIST compared conventional Raman spectra of CVD diamond (obtained with visible light excitation) with Fourier Transform Raman (FT-Raman) spectra obtained with an excitation wavelength of 1.06 μm. While no significant difference was found between the two types of spectra obtained from a type IIa single crystal diamond, radical spectral differences were observed between the two types of sjaectra from CVD diamond. CVD diamond specimens exhibiting extremely clean Raman spectra in the visible show a large number of spectral features in the infrared region. Thus, FT-Raman spectroscopy appears to be a much more sensitive probe of diamond quality than visible Raman spectroscopy. Of note was a pronounced spectral feature in the FT-Raman spectrum of CVD diamond that was not observed in the visible Raman spectrum.

## 4. Standardizing Thermal Conductivity Measurements

Four presentations were made during this session.

J. Graebner of AT&T reported the results of thermal conductivity measurements on an isotopically enriched CVD diamond plate [amount of-substance (or atom number) fraction of ^12^C, 0.055%]. At room temperature, the in-plane thermal conductivity was 22 W/(cm · K) and the perpendicular-to-plane thermal conductivity was 26 W/(cm · K). Not only are these values higher than any previously reported for CVD diamond, but the perpendicular value is higher than that for the best natural single crystal diamond with the normal abundance of ^13^C. An analysis of the temperature dependence of the thermal conductivity was consistent with a reduction in point-defect scattering of phonons in the isotopically enriched CVD diamond. This would account for the higher thermal conductivity.

O. Käding of Daimler-Benz described thermal conductivity measurements using photothermal displacement spectroscopy at transient thermal gratings (PDS-TTG), In this method, a polished diamond surface is heated by two pulsed laser beams that interfere to form a transient periodic heating pattern (grating) on the specimen surface. The relaxation time for the heating pattern to dissipate is governed by the period of the grating and the lateral thermal diffusivity. The relaxation time is determined by measuring the change in the deflection angle of a cw laser beam that is reflected from an undulation in the sample surface caused by the heating. The depth dependence of the thermal diffusivity near the surface can be determined by varying the grating period.

A. Feldman of NIST presented the results of round robin measurements of thermal conductivity. Measurements where performed by six laboratories on ten specimens provided by four manufacturers. Three geometries were provided: squares, disks, and long/narrow strips. Most laboratories could not test every specimen because each measurement procedure usually required a specific specimen geometry. Typically, the highest value and the lowest value for a given specimen differed by a factor of two. However, such a comparison is not necessarily meaningful because the thermal conductivities of the specimens were inhomogeneous and anisotropic. Each of the techniques sampled the specimen properties in different ways so that different results were to be expected. However, even measurements that should have yielded similar results showed variations up to 80%.

G. Lu of Norton Diamond Film presented the results of a survey that had been sent to users of CVD diamond for thermal management inquiring about their need for a standardized measurement method. When asked if only one type of measurement could be performed, what would they prefer it to be, a clear majority preferred in-plane thermal conductivity rather than a perpendicular-to-plane measurement or an approximate average of the two. A clear majority also chose ± 10% as the minimum uncertainty needed. There was a strong preference for a less accurate, quality control tool that would be used on every lot rather than a more accurate measurement that sampled every fifth or tenth lot. However, there was no consensus on other questions such as: was it preferable to determine both the in-plane and perpendicular-to-plane thermal conductivity on an occasional basis; or, should the thermal conductivity of every lot be measured in one direction.

## 5. Characterizing Stress, Strain, and Fracture

Recent advances in the art of producing CVD diamond now make it possible to obtain free-standing millimeter-thick deposits that have elastic, optical, and thermal properties comparable to those of type-IIa natural diamond crystals. These deposits, however, exhibit fracture strengths that are substantially lower than anticipated judging from the reported strength values of single crystals (natural and synthetic) or polycrystalline high-temperature/high-pressure produced diamond compacts. Since many of the potential applications of CVD diamond, for example infrared transmitting windows or domes, critically depend on the ability to withstand stresses generated in a thermal shock environment, it has become essential to properly assess the fracture behavior of this material. Furthermore, the production of thick CVD diamond plates is often hindered by cracking of the deposit, which suggests that the CVD process gives rise to internal stresses that may initiate crack propagation. The purpose of this session was to explore how a proper description of the development of strains and stresses in CVD diamond may help us understand its strength and fracture characteristics. In this regard, it has been established that the deposition process itself generates highly localized stress concentrations at the grain boundaries, thus weakening the structure. These stress concentrations appear to result from the intrinsic elastic anisotropy, from the formation of high-order twins boundaries, and (perhaps) from the defects associated with the presence of residual hydrogen impurities. Still, the issue of how to control process-induced stresses, in effect, establishing the relationship between growth conditions and state of stress, has not yet been resolved. Ten presentations at this session addressed these questions.

Evidence of internal strains in CVD diamond films was first reported by Japanese workers [1] based on Raman spectroscopy work. Y. Sato of the National Institute for Research in Inorganic Materials indicated that measurements performed on films 10 μm to 30 μm thick deposited on silicon showed peak-position displacements up to 3 cm^−1^ in addition to substantial line broadening, which points to inhomogeneous strain distributions. Microscopic birefringence patterns seen on polished surfaces also demonstrate that, besides gradual stress variations between top and bottom layers, there were steep stress gradients, perhaps up to 5 GFa/μm, within regions comparable to the grain size. At Norton, K. Gray obtained Raman spectra at various locations on an optical quality 0.5 mm thick CVD diamond bar uniaxially stressed in a three-point bending fixture and observed that the Raman peak shifts in accord with the hydrostatic pressure coefficient, 2.9cm^−1^/GPa, previously recorded for single crystals. Because of the large beam spot-size (30 μm), the Norton spectra do not provide the resolution required to detect highly localized stresses as was done at the Naval Research Laboratory (NRL). J. Butler reported that the micro-Raman instrument used at NRL, which operates at a wavelength of 5)4.5 nm, provides spatial resolutions as low as 1 μm in addition to exceptional spectral resolution. Thus, the stress can be assessed not only in terms of the shifting and the broadening of the diamond Raman line but also from the splitting of the line that occurs due to the lifting of the longitudinal-optic/transverse-optic phonon degeneracy. In accord with the NIRIM observations, this important work leads to the conclusion that residual stresses in CVD diamond films are highly localized and exhibit a spatial distribution that correlates well with surface morphological features; peak stresses exceeding 3 GPa have been recorded.

At this point, it becomes desirable to examine what causes the stress concentrations, and three papers addressed that issue.

S. Kurtz and collaborators at the Pennsylvania State University are making use of the Poisson-Voronoi tessellation model, in conjunction with finite-element methods, to calculate both the effective elastic modulus of random aggregates of diamond grains and the microstresses that develop at the periphery of individual grains in textured diamond deposits. Their result for Young’s modulus was shown to be in remarkable agreement with the value obtained elsewhere [2] upon using the Hershey-Kröner-Eshelby averaging procedure. More importantly, these numerical simulations show that, near grain triple points in textured thin films, there are very large stress concentrations, or stress singularities, which derive from the elastic anisotropy of the diamond lattice and depend on the degree of preferred orientation.

On a more visual level, D. Shechtman of the Technion (at present, guest scientist at NIST and NRL) considered the impact of low-order twin boundaries that evolve during the deposition of CVD diamond and that play a role in providing suitable nucleation sites. The intersection of these low-order twin boundaries leads to the formation of higher-order twin boundaries that may contribute to creating locally stressed regions. The columnar structure of CVD diamond, specifically, the presence of grain boundaries as well as twin boundaries, thus can provide a direct fracture path and, therefore, contribute to lowering the fracture resistance.

Very comprehensive investigations of the relationships between growth conditions, microstructure, and residual stresses in microwave-plasma CVD diamond deposits were reported on by A. Marker of Rockwell International. The characterization tools he uses to examine microstresses include angle-resolved x-ray diffraction, channeling electron microscopy, and stimulated fluorescence. One of the conclusions of his work is that even if there are wide fluctuations in local stress levels, the average residual bulk stress appears to be rather insignificant with the growth surface generally under tension and the nucleation surface under minor compression.

The latter work corroborates some of the results of R. Hallock and C. Klein of Raytheon, who performed wafer-curvature experiments on diamond/silicon laminates for the purpose of characterizing the average intrinsic strain of CVD diamond as a function of the diamond thickness and deposition temperature. In this connection, they emphasized that if the proper thin-film conditions are not satisfied, commonly used formulas for obtaining the coating stress from the wafer curvature involve semi-empirical approximations of questionable validity. The model they are using to interpret their data is based upon a general theory of elastic interactions in multi-layer laminates [3] that assumes isotropic relationships in the layer planes, in other words, texture-free potycrystalline deposits on (111) or (100) Si substrates. Errors originate primarily from some unavoidable uncertainty in assessing the growth temperature and, hence, in determining the thermal mismatch strain; in addition, Si creep may cause problems at growth temperatures in excess of ~950°C. For these reasons, wafer-curvature experiments were also carried out with diamond on diamond systems, which not only eliminates the thermal mismatch but yields direct evidence on intrinsic strains since the wafer curvature is related to the coating strain through a very simple equation. In both types of experiments (diamond on silicon as well as diamond on diamond), the average process-induced strain was found to be relatively small but strongly dependent upon the deposition temperature, the sense of the strain changing from compressive at lower temperatures to tensile at temperatures above 1030°C.

C. Klein of C.A.K Analytics commented that in evaluating the fracture-strength of CVD diamond it is essential to keep in mind that the crack propagation mechanism appears to be controlled by surface flaws. Consequently, the stress at failure cannot be considered indicative of an intrinsic fracture strength because it obeys a probability law. In effect, the failure probability at a given applied stress obeys the Weibull distribution. An analysis of fracture-strength measurements performed on CVD diamond films [4] made by hot filament CVD, 10 μm to 100 μm thick, yielded a characteristic strength of ~485 MPa at the growth surface and 1030 MPa at the nucleation surface.

J. Mecholsky of the University of Florida confirmed that polycrystalline diamond indeed fails from the surface and outlined current approaches to describing the fracture behavior, which include fracture mechanics considerations, the fractal geometry description of a fractured surface, and molecular dynamics modeling. Fracture mechanics experiments were used to determine the critical fracture energy and the fracture toughness. Using the characteristic strength values reported above, one can estimate the flaw sizes to range from 30 μm at the nucleation surface to 150 μm at the growth surface; these dimensions are roughly equal to the estimated grain sizes at these surfaces. Also of interest is Mecholsky’s observation, using the sin^2^(ψ) x-ray diffraction technique, that the stress pattern in diamond coatings deposited on silicon shows evidence of growth stresses, i.e., stresses that cannot be accounted for by thermal expansion mismatch alone. This conclusion is in accordance with the wafer-curvature work done at Raytheon.

Finally, M. Drory of Crystallume obtained a fracture toughness value of 5.3 MPa · m^1/2^ for CVD plates thicker than 200 μm that had been subjected to a uniaxial tensile stress. This value, which is typical of high-strength ceramic materials, agrees with Drory’s earlier value derived from indentation testing but appears to be lower than values obtained by other workers and may reflect specimento-specimen variations in microstructural features. How such variations would affect the fracture toughness is not really understood.
